# A New Remedy for Gout and Rheumatism

**Published:** 1846-03-01

**Authors:** 


*A new remedy for Gout and Rheumatism.*—Doct. T. H. Buckler, of Baltimore, in the Jan. No. of the Am. Jour. Med. Sciences, propounds a new remedy for Gout and Rheumatism, viz.—the phosphate of Ammonia.

Dr. B. was led to the use of this remedy by hypothetical reasoning, based upon the chemical composition of this salt, and the supposed pathology of these diseases. His views are briefly set forth in the following extract: "Taking into account these two prominent facts, namely, the excess of lithic acid found in the urine at the period of convalescence from an attack of acute gout or Rheumatism, and the subsequent deposit of soda and lime in the white tissues, it occurred to me that during the existence of these diseases, the lithic acid might exist in the blood in a state of combination with soda and lime in the form of insoluble compounds, which the kidneys and skin refuse to eliminate. If, then, any agent could be found capable of decomposing the lithates of soda and lime existing in the blood, and of forming in their stead two soluble salts, which would be voided by the kidneys and skin, we should thereby get rid of the excess of fibrin in the blood, the symptomatic fever, and the gouty and rheumatic inflammation, wherever seated, which have been excited by the presence of these salts. It occurred to me that *phosphate of Ammonia* might be the agent provided it could be given in doses sufficient to answer the end without producing any unpleasant symptoms. If our theory were true, phosphate of Ammonia seemed to be the proper re-agent, for it would form in place of the insoluble lithate of soda, two soluble salts, the phosphate of soda which is remarkably soluble, and the lithate of ammonia which is also soluble, and both capable of being readily passed by the skin and kidneys. The excess of uric acid would thus be got rid of in the form of lithate of ammonia; and the soda, floating in the round of the circulation (instead of being deposited, as it were, like an alluvial formation in the substance of the fibrous and cartilaginous tissues,) would be taken up by the phosphoric acid and eliminated from the circulation."

Thirteen cases are reported in which the remedy appeared to exert considerable potency in curing these diseases. Five of the cases were observed by Dr. Buckley, five by Dr. Frick, and three by Dr. Power. The formulas in these cases varied from half an ounce to an ounce of the salt dissolved in six ounces of distilled water, the dose being a table spoonful every six hours. The effect of the remedy upon the urinary secretion was to cause the lithic acid to disappear if it were previously present; and if it had not been present, the urine continued limpid and free from precipitate of any kind through convalescence.

Dr. B. does not propose it as an exclusive remedy, but to be employed in conjunction with bleeding, general and topical, anodynes, &c., according to the symptoms.

Dr. B. has also prescribed it in chronic rheumatism pretty extensively, and with much more relief than from any of the usual remedies. He also feels much confidence that it will be found to be the best agent for dissolving the uric acid calculus; but he has not had any opportunity of testing its efficacy in this affection.

We have read this communication with much interest. It seems now pretty well established, that the pathology of gout and rheumatism involves especially a morbid condition of the blood. The abundant secretion of lithic acid, particularly as coincident with convalescence, and the deposit of the lithate of soda in the fibrous tissues constituting the chalky concretions of gout, would indicate that the undue formation of this acid was the chief humoral difficulty, giving rise to the febrile disturbance, and, perhaps, by some unknown mode of action, to the predominance of fibrin which is the characteristic of the acute stage of the disease. This view of the pathology is sustained by the mode of action of those remedies which have been found most efficaceous—such as mercurials, colchicum, nitrate of potash and the iodide of potassium. They all exert their influence especially on the emunctories—particularly the skin and urinary organs. Mere *a priori* reasoning will not, however, suffice in therapeutics; and the writer accordingly has collected facts before announcing his supposed discovery. The number of facts must be extended before any positive conclusions can be deduced. This we hope will be done, more especially since these diseases are among the *opprobria* of our science.

We commend the article to the perusal of our readers from the general views which it contains. In common with the writer, we look forward to practical results from the present application of analytical chemistry to the fluids of the body in health and disease, which will very materially affect the condition of pathology and therapeutics. We believe the time will come when forms of disease now intelligible only in their effects will be intimately understood, the operation of remedies whose virtues have been ascertained by empirical experience, explained, and medicine become a truly rational art. If we do not anticipate too much from organic chemistry, the ultra vitalists and solidists will shortly be left far behind in the progressive advancement of our science.

